# A physical activity program dedicated to adolescents

**DOI:** 10.1186/1824-7288-40-S1-A24

**Published:** 2014-08-11

**Authors:** Maria Cristina Maggio

**Affiliations:** 1 Universitary Department Pro.S.A.M.I. “G. D’Alessandro”, University of Palermo, Italy

## 

Adolescence is a life stage in which the development of individuality and self-identity occurs.

The recent study of the SIP evaluated the life style in adolescents, highlighting the low participation to continuative sports programs and the high incidence to sports drop out, interesting more than 30% of adolescents. These problems are partially linked to low compliance of adolescents to coaches training, partially to the request of a sport close to adolescents requirements.

However sports participation is beneficial for physical and psychological development of adolescents. Sports programs promote responsible social behaviours and greater academic success, confidence in personal physical abilities, appreciation of personal health and fitness and strong social bonds with individuals and institutions. Adolescents involved in physical activities fare better academically, have higher relationship skills, are more team-oriented and are healthier as determined by fitness standards.

For these reasons the program of the physical activity dedicated to the adolescent needs a personal evaluation of own desires and personal abilities, with a daily promotion of an active life style, preferring group activities (to go to school and to come back home by foot or by bike with friends, to walk for familial needs, etc.). The sport choice must be carried out together with them, in order to guarantee a four times a week physical activity, in conformity with scholastic needs. Once a week they need to have special physical activities in accordance to familial requests.

The skills of this program are: the education to a maintained physical assignment; the prevention of social deviation and/or alcohol, smoke, drugs dependence; the education to roles and rules, responsibilities, loyalty, cooperation, friendship.

